# Association between electronic cigarette use and prediabetes, diabetes, and insulin resistance: a systematic review and meta-analysis

**DOI:** 10.1007/s11739-026-04430-x

**Published:** 2026-06-13

**Authors:** Yusuff Adebayo Adebisi, Davide Campagna, Antonio Ceriello, Anas Ali Alhur, Najim Z. Alshahrani, Anoop Misra, Abdul Basit, Cristina Russo, Tadej Battelino, Noel Somasundaram, Muhammad Yazid Jalaludin, Yoshifumi Saisho, Magdalena Walicka, Venera Tomaselli, Dianna J. Magliano, Othmar Moser, Joanna Mitri, Roger Chen, Riccardo Polosa

**Affiliations:** 1 College of Social Sciences, University of Glasgow, 40 Bute Gardens, Glasgow, G12 8RT Scotland, UK; 2 Department of Clinical and Experimental Medicine, University of Catania, Catania, Italy; 3 Nuffield Department of Population Health, University of Oxford, Oxford, UK; 4 Center of Excellence for the Acceleration of Harm Reduction (CoEHAR), University of Catania, Catania, Italy; 5 IRCCS MultiMedica, Milan, Italy; 6 Department of Health Informatics, College of Public Health and Health Informatics, University of Hail, Hail, Saudi Arabia; 7 Department of Family and Community Medicine, Faculty of Medicine, University of Jeddah, Jeddah, Saudi Arabia; 8 Diabetes Foundation, New Delhi, India; 9 National Diabetes, Obesity and Cholesterol Foundation (N-DOC), New Delhi, India; 10 Fortis C-DOC Centre for Excellence for Diabetes, Metabolic Disease, and Endocrinology, New Delhi, India; 11 Indus Diabetes & Endocrinology Center, Indus Hospital & Health Network, Karachi, Pakistan; 12 Ashford and Saint Peter’s Hospitals NHS Foundation Trust, Chertsey, UK; 13 University Medical Center Ljubljana, and Faculty of Medicine, University of Ljubljana, Ljubljana, Slovenia; 14 Diabetes and Hormone Center, Colombo, Sri Lanka; 15 Department of Paediatrics, Faculty of Medicine, Universiti Malaya, Kuala Lumpur, Malaysia; 16 Saisho Diabetes Clinic, Tokyo, Japan; 17 Clinical Department of Internal Medicine, Endocrinology and Diabetology, National Medical Institute of the Ministry of the Interior and Administration, Warsaw, Poland; 18 Mossakowski Medical Research Institute, Polish Academy of Sciences, Warsaw, Poland; 19 Department of Economics and Business, University of Catania, Catania, Italy; 20 School of Public Health and Preventive Medicine, Monash University, Melbourne, VIC Australia; 21 Exercise Physiology, Training & Training Therapy Research Group, Institute of Human Movement Science, University of Graz, Sport & Health, Graz, Austria; 22 Division of Endocrinology and Diabetology, Interdisciplinary Metabolic Medicine Trials Unit, Medical University of Graz, Graz, Austria; 23 Department of Medicine, Harvard Medical School, Boston, MA 02115 USA; 24 Vincent’s Hospital, Sydney, Australia

**Keywords:** Electronic cigarettes, E-cigarettes, Vaping, Diabetes mellitus, Prediabetes, Insulin resistance, Meta-analysis, Systematic review

## Abstract

Electronic cigarette (e-cigarette) use has grown rapidly worldwide, yet metabolic health consequences, particularly glycaemic outcomes, remain poorly understood. This systematic review and meta-analysis synthesises the available epidemiological evidence on the association between e-cigarette use and diabetes mellitus, prediabetes, and insulin resistance. We conducted a systematic search of PubMed, Scopus and Embase from inception through December 2025, following PRISMA 2020 guidelines. Studies reporting effect estimates for the association between e-cigarette use and diabetes, prediabetes, or insulin resistance in adult populations were eligible. Random-effects meta-analyses were performed, stratified by outcome and exposure category (current exclusive e-cigarette users, dual users, and former e-cigarette users versus never users). Heterogeneity was assessed using I^2^ statistics and Cochran’s Q test. Ten studies (9 cross-sectional, 1 prospective cohort; combined participants exceeding 2.8 million) met inclusion criteria; 8 cross-sectional studies contributed to quantitative pooling, while one cross-sectional study and the prospective cohort were narratively synthesised. For prediabetes, pooled ORs versus never users were 1.34 (95% CI 1.01–1.77; *I*^2^ = 74.2%) for current exclusive users, 1.26 (1.06–1.49; *I*^2^ = 75.4%) for dual users, and 1.13 (1.06–1.20; *I*^2^ = 0%) for former users. For diabetes, only dual users showed a significant elevation (OR 1.23; 1.01–1.49; *I*^2^ = 77.4%); current exclusive (OR 1.04; 0.93–1.16; *I*^2^ = 45.1%) and former users (OR 1.11; 0.80–1.54; *I*^2^ = 31.6%) did not differ from never users. For insulin resistance, current exclusive e-cigarette users versus never users had an OR of 1.37 (95% CI 1.06–1.77; *I*^2^ = 0%). The sole prospective cohort study found no significant association between exclusive e-cigarette use and incident type 2 diabetes (HR 0.88; 95% CI 0.66–1.16). Neither cross-sectional nor prospective evidence linked exclusive e-cigarette use to diabetes; significant associations were confined to dual users. Elevated odds of prediabetes and insulin resistance among exclusive users were observed cross-sectionally but cannot support causal claims. Large prospective studies with longer follow-up, objective verification of product exposure status (for example, biomarkers distinguishing exclusive vaping from combustible tobacco use), and standardised laboratory-based glycaemic endpoints are needed.

**Supplementary Information:**

The online version contains supplementary material available at 10.1007/s11739-026-04430-x.

## Introduction

Electronic cigarettes (e-cigarettes) have become widely used nicotine delivery products globally, with an estimated 129 million users in 2025 [1]. Developed and promoted as lower risk alternatives to conventional cigarettes, e-cigarettes produce an inhaled aerosol by heating a liquid that commonly contains nicotine, propylene glycol, vegetable glycerine, and flavourings [2, 3]. Although they are substantially less toxic than combustible cigarettes [4–6] [50], debate continues regarding their role in smoking cessation and tobacco harm reduction [7]. Increasing attention has also focussed on their potential independent cardiovascular and metabolic effects [8, 44].

Diabetes mellitus is a major global public health problem, affecting more than 500 million adults worldwide and projected to rise to 783 million by 2045 [9, 10]. In many cases, type 2 diabetes is preceded by prediabetes, an intermediate metabolic state marked by impaired fasting glucose and/or impaired glucose tolerance. Prediabetes is estimated to affect 541 million people globally [9, 10]. Insulin resistance, which plays a central role in the development of both prediabetes and diabetes, is increasingly understood as a systemic process that may be shaped by environmental exposures, including tobacco smoke [11, 12].

The association between conventional cigarette smoking and diabetes is well documented [13]. A major meta-analysis found that active smoking was associated with an approximately 37% higher risk of diabetes, with evidence of a dose–response relationship [14]. Although this association is well established, the exact biological pathways through which smoking increases the risk of incident diabetes are not yet fully resolved. Proposed mechanisms include smoking-induced chronic inflammation, reduced insulin sensitivity, and impairment of pancreatic β-cell function [15]. These hypotheses are biologically plausible given the complex composition of cigarette smoke, which contains more than 7,000 chemicals, including carbon monoxide, polycyclic aromatic hydrocarbons, heavy metals, and oxidative gases. Many of these constituents may contribute to insulin resistance through inflammatory pathways, β-cell damage, and hormonal disruption. Smoking has also been linked to altered circulating sex hormones, particularly in women, including higher levels of testosterone, estradiol, and sex-hormone-binding globulin [16, 17]. Even so, separating the metabolic effects of nicotine itself from the wider toxic effects of combustion remains difficult. This distinction is especially important when considering e-cigarettes, which deliver nicotine in the absence of combustion. Nicotine itself has plausible metabolic effects relevant to diabetes pathogenesis. Experimental evidence indicates that nicotine may impair insulin signalling and promote peripheral insulin resistance [45–47], and may adversely affect pancreatic β-cell viability and mitochondrial function [48, 49]. These mechanisms provide biological plausibility for examining whether nicotine-containing aerosol exposure could influence prediabetes or diabetes risk, even in the absence of combustion.

Notwithstanding this biological plausibility, the epidemiological evidence on e-cigarette use and glycaemic outcomes has not yet been systematically synthesised in a comprehensive way. No previous meta-analysis has evaluated prediabetes, diabetes, and insulin resistance as distinct outcomes while also separating mutually exclusive exposure categories. Distinguishing current exclusive e-cigarette users, dual users, and former users is important for determining whether observed associations reflect e-cigarette exposure itself rather than concurrent combustible cigarette smoking. This systematic review and meta-analysis was therefore conducted to identify, critically appraise, and quantitatively synthesise the available observational evidence on the association between e-cigarette use and prediabetes, diabetes, or insulin resistance in adult populations. In doing so, we aimed to inform clinical interpretation, public health discussion, and future research priorities.

## Methods

This systematic review and meta-analysis was conducted in accordance with the Preferred Reporting Items for Systematic Reviews and Meta-Analyses (PRISMA) 2020 guidelines [18]. The completed PRISMA 2020 checklist is provided as Supplementary Material. This review was not prospectively registered in PROSPERO, which we acknowledge as a methodological limitation.

### Search strategy

A comprehensive literature search was conducted in PubMed, Scopus, and Embase from database inception through December 2025. The search strategy combined controlled vocabulary (Medical Subject Headings [MeSH] in PubMed and Emtree subject headings in Embase) with free-text keywords across two conceptual domains: (1) metabolic outcomes, including clinical diagnoses (diabetes mellitus, prediabetes) and laboratory-based glycaemic markers (impaired glucose tolerance, impaired fasting glucose, insulin resistance, HOMA-IR, TyG index, hyperinsulinaemia, and metabolic syndrome); and (2) e-cigarette exposure, including electronic cigarettes, e-cigarettes, vaping, and electronic nicotine delivery systems. Boolean operators were used to combine terms within and between domains, and truncation operators were applied where appropriate. The complete search strategies for each database are provided in Table 1.

**Table 1:** Database search strategies (searched from inception to December 2025)

Database	Search term
PubMed	(“Diabetes Mellitus, Type 2”[MeSH Terms] OR “Prediabetic State”[MeSH Terms] OR “Insulin Resistance”[MeSH Terms] OR “Metabolic Syndrome”[MeSH Terms] OR “type 2 diabetes”[Title/Abstract] OR “T2DM”[Title/Abstract] OR “prediabetes”[Title/Abstract] OR “pre-diabetes”[Title/Abstract] OR “impaired glucose tolerance”[Title/Abstract] OR “impaired fasting glucose”[Title/Abstract] OR “insulin resistance”[Title/Abstract] OR “HOMA-IR”[Title/Abstract] OR “homeostatic model assessment”[Title/Abstract] OR “fasting insulin”[Title/Abstract] OR “hyperinsulinemia”[Title/Abstract] OR “TyG index”[Title/Abstract]) AND (“Electronic Nicotine Delivery Systems”[MeSH Terms] OR “E-Cigarette Vapor”[MeSH Terms] OR “Vaping”[MeSH Terms] OR “electronic cigarette*”[Title/Abstract] OR “e-cigarette*”[Title/Abstract] OR “e cig*”[Title/Abstract] OR “vaping”[Title/Abstract] OR “vaper*”[Title/Abstract] OR “electronic nicotine delivery system*”[Title/Abstract])
Embase	#1 Metabolic Outcomes (‘type 2 diabetes mellitus’/exp OR ‘prediabetes’/exp OR ‘insulin resistance’/exp OR ‘metabolic syndrome’/exp OR ‘type 2 diabetes’:ti,ab,kw OR ‘T2DM’:ti,ab,kw OR prediabetes:ti,ab,kw OR ‘impaired glucose tolerance’:ti,ab,kw OR ‘impaired fasting glucose’:ti,ab,kw OR ‘insulin resistance’:ti,ab,kw OR ‘HOMA-IR’:ti,ab,kw OR ‘homeostatic model assessment’:ti,ab,kw OR ‘fasting insulin’:ti,ab,kw OR hyperinsulinemia:ti,ab,kw OR ‘TyG index’:ti,ab,kw) #2 E-cigarette Exposure (‘electronic cigarette’/exp OR ‘vaping’/exp OR ‘electronic nicotine delivery system’/exp OR ‘e-cigarette*’:ti,ab,kw OR ‘e cig*’:ti,ab,kw OR vaping:ti,ab,kw OR vaper*:ti,ab,kw OR ‘electronic nicotine delivery system*’:ti,ab,kw) #3 Final: #1 AND #2
Scopus	(TITLE-ABS-KEY (“type 2 diabetes” OR T2DM OR prediabetes OR “pre-diabetes” OR “impaired glucose tolerance” OR “impaired fasting glucose” OR “insulin resistance” OR “HOMA-IR” OR “homeostatic model assessment” OR “fasting insulin” OR hyperinsulinemia OR “TyG index” OR “metabolic syndrome”)) AND (TITLE-ABS-KEY (“electronic cigarette*” OR “e-cigarette*” OR “e cig*” OR vaping OR vaper* OR “electronic nicotine delivery system*” OR ENDS))

### Eligibility criteria

Studies were included if they met the following criteria: (1) observational design (cross-sectional, case–control, or cohort); (2) adult participants (≥ 18 years); (3) e-cigarette use as the exposure of interest, with a clearly defined never-user or non-user reference group; (4) at least one of the following outcomes: diabetes mellitus, prediabetes (including impaired fasting glucose or impaired glucose tolerance), or insulin resistance (measured by HOMA-IR, TyG index, or equivalent validated indices); and (5) reporting of adjusted effect estimates (odds ratios, hazard ratios, or risk ratios) with 95% confidence intervals, or sufficient data to calculate these. Studies were excluded if they (1) were animal or in vitro experiments; (2) enrolled only paediatric populations (< 18 years); (3) did not separate e-cigarette users from conventional cigarette users; or (4) were case reports, editorials, commentaries, or conference abstracts without sufficient data. No restrictions were placed on diabetes subtype classification; studies reporting self-reported physician-diagnosed diabetes or laboratory-defined diabetes (based on fasting plasma glucose, HbA1c, or medication use) were eligible regardless of whether the specific diabetes type was specified.

### Study selection and data extraction

Two reviewers independently screened titles and abstracts, followed by full-text review of potentially eligible records. Discrepancies were resolved through discussion and consensus, with a third reviewer consulted where necessary. Data extraction was conducted using a standardised, piloted form. Extracted variables included first author, publication year, country, data source, study period, sample size, participant demographics, exposure categories (including assessment of mutual exclusivity), outcome definitions and measurement methods, covariates included in adjusted models, and adjusted effect estimates with corresponding 95% confidence intervals. Where multiple adjusted models were reported, the most comprehensively adjusted estimate was extracted. For studies reporting multiple exposure categories (e.g. current exclusive use, former use, and dual use), comparisons against the never-user reference group were extracted separately for subgroup analyses. Each study contributed at most one effect estimate to any single exposure–outcome meta-analysis.

### Risk of bias and certainty of evidence assessment

The methodological quality of included studies was assessed using the Newcastle–Ottawa Scale (NOS), adapted for cross-sectional studies where applicable. The NOS evaluates studies across three domains: selection of participants, comparability of groups, and ascertainment of exposure and outcome. Each study was assigned a score out of a maximum of 9 stars for cohort studies or 10 stars for cross-sectional studies, with higher scores indicating lower risk of bias. Two reviewers independently assessed study quality, with disagreements resolved by discussion.

The certainty of evidence for each outcome was evaluated using the Grading of Recommendations, Assessment, Development and Evaluations (GRADE) framework, with observational evidence starting at low certainty and assessed across five domains: risk of bias, inconsistency, indirectness, imprecision, and publication bias.

### Statistical analysis

All analyses were conducted in Stata 18 (StataCorp, College Station, TX) using the *meta* suite of commands. Adjusted odds ratios and their 95% confidence intervals were the primary effect measure. Effect estimates were log-transformed for analysis and back-transformed (exponentiated) for presentation. Given the anticipated clinical and methodological heterogeneity across studies, random-effects models using restricted maximum likelihood (REML) estimation were employed throughout.

Separate meta-analyses were conducted for three outcomes: prediabetes, diabetes, and insulin resistance. Within each outcome, subgroup analyses were performed according to the exposure comparison: (a) current exclusive e-cigarette users versus never users, (b) dual users (concurrent e-cigarette and conventional cigarette use) versus never users, and (c) former e-cigarette users versus never users. Between-subgroup differences were evaluated using the *Q*^b^ test for heterogeneity.

Where individual studies reported multiple comparisons against the same reference group (e.g. current, dual, and former e-cigarette users versus never users), each estimate was included in the relevant exposure subgroup analysis. It is acknowledged that estimates drawn from the same study are not fully independent, as they share a common reference group; however, these estimates were never combined within the same subgroup stratum, and this approach is consistent with standard practice in meta-analyses stratifying by exposure category. Accordingly, each study contributed at most one effect estimate to any single exposure–outcome meta-analysis.

Statistical heterogeneity was quantified using the *I*^2^ statistic and Cochran’s *Q* test. *I*^2^ values of 25%, 50%, and 75% were interpreted as low, moderate, and high heterogeneity, respectively. Formal assessment of publication bias and small-study effects (e.g. funnel plots or regression-based asymmetry tests such as Egger’s test) was not performed because each pooled comparison included fewer than 10 studies, for which these methods have low power and limited interpretability. A two-tailed *p*-value < 0.05 was considered statistically significant for all tests.

## Results

### Study selection

**Fig. 1:**
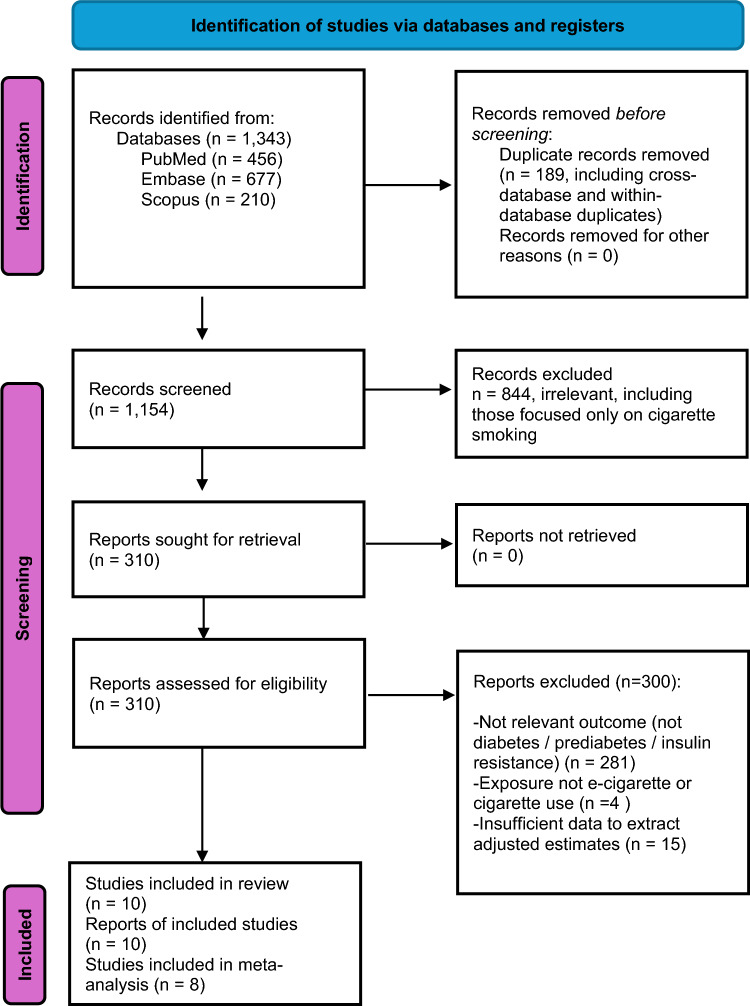
PRISMA 2020 flow diagram of study identification, screening, eligibility assessment, and inclusion

Ten studies met all inclusion criteria and were included in the qualitative synthesis of data [19–28] (Fig. 1). Of these, eight cross-sectional studies reported adjusted odds ratios and contributed to the quantitative meta-analyses. Two studies were narratively described [25, 28].

### Study characteristics

The 10 included studies [19–28] comprised nine cross-sectional analyses and one prospective cohort study, published between 2019 and 2025. The combined sample exceeded 2.8 million adult participants. Six studies were conducted in the United States, utilising nationally representative datasets including the Behavioural Risk Factor Surveillance System (BRFSS; *n* = 3) [19, 20, 23], the National Health and Nutrition Examination Survey (NHANES; *n* = 2) [21, 25], and the All of Us Research Program (*n* = 1) [28]. Three studies were from South Korea, using the Korea Community Health Survey (KCHS) [22] and the Korea National Health and Nutrition Examination Survey (KNHANES) [24, 27]. One study utilised data from the Scottish Health Survey [26]. Study characteristics are summarised in Table 2.

**Table 2:** Characteristics of included studies (*n* = 10)

Study	Country	Data source	Design	*N*	Outcome	Key estimates
Atuegwu et al., 2019 [19]	USA	BRFSS 2016–2017	Cross-sectional	154,404	Self-reported prediabetes	Current e-cig vs never: OR 1.97 (1.25–3.10); Former e-cig vs never: OR 1.07 (0.84–1.37)
Zhang et al., 2022 [20]	USA	BRFSS 2016–2018	Cross-sectional	600,046	Self-reported prediabetes	Current e-cig vs never: OR 1.54 (1.17–2.03); Former e-cig vs never: OR 1.12 (1.05–1.19); Dual user vs never: OR 1.14 (0.97–1.34)
Cai et al., 2023 [21]	USA	NHANES 2013–2018	Cross-sectional	5101	Lab-defined DM, prediabetes, HOMA-IR (Insulin resistance)	Prediabetes: current e-cig only vs never OR 1.14 (0.68–1.92); former e-cig only vs never OR 1.14 (0.84–1.54); dual vs never OR 0.80 (0.46–1.39) Diabetes: current e-cig only vs never OR 0.73 (0.29–1.85); former e-cig only vs never OR 1.45 (0.84–2.51); dual vs never OR 0.74 (0.26–2.14) Insulin resistance (HOMA-IR Q3 vs Q1): current e-cig only vs never OR 1.33 (0.77–2.30)
Jeong & Kim, 2024 [22]	Korea	KCHS 2021–2022	Cross-sectional	460,603	Self-reported diabetes	E-cig only vs non-smoker: OR 1.15 (1.01–1.31) Dual user vs never: OR 1.39 (1.22–1.58)
Neupane et al., 2025 [23]	USA	BRFSS 2020–2022	Cross-sectional	1,285,783	Self-reported diabetes and prediabetes	Prediabetes: current e-cig only vs never OR 1.07 (1.03–1.11); dual vs never OR 1.28 (1.23–1.33) Diabetes: current e-cig only vs never OR 1.01 (0.98–1.04); dual vs never OR 1.09 (1.07–1.12)
Lim et al., 2025 [24]	Korea	KNHANES 2019–2020	Cross-sectional	4404	IR (TyG index); Insulin resistance	E-cig vs never: OR 1.38 (1.03–1.84)
Orimoloye et al., 2019 [25]	USA	NHANES 2013–2016	Cross-sectional	3989	HOMA-IR/GTT (Insulin resistance)	HOMA-IR: Sole e-cig vs non-users: β 0.20 (− 0.09 to 0.49); Dual vs non-users: β − 0.13 (− 0.43 to 0.16) GTT: Sole e-cig vs non-users: β − 0.05 (− 0.21 to 0.11); Dual vs non-users: β − 0.12 (− 0.24 to − 0.004)
Adebisi et al., 2025 [26]	Scotland	SHeS 2017–2021	Cross-sectional	17,854	Self-reported diabetes	E-cig only vs never: OR 0.81 (0.53–1.22) Dual vs never: OR 1.49 (0.94–2.37) Former e-cig vs never: OR 1.00 (0.78–1.29)
Kim et al., 2022 [27]	Korea	KNHANES 2014–2018	Cross-sectional	22,385	Prediabetes (HbA1c)	Dual vs never: OR 1.57 (1.29–1.92) Former e-cig vs never: OR 1.54 (1.11–2.14)
Erhabor et al., 2025 [28]	USA	All of Us (~ 4 yr)	Prospective cohort	249,190	Incident diabetes	Exclusive e-cig vs never: HR 0.88 (0.66–1.16)

*BRFSS* Behavioural Risk Factor Surveillance System, *NHANES* National Health and Nutrition Examination Survey, *KCHS*, Korea Community Health Survey, *KNHANES* Korea National Health and Nutrition Examination Survey, *SHeS* Scottish Health Survey, *HbA1c* glycated haemoglobin, *HOMA*-*IR* Homeostatic Model Assessment of Insulin Resistance, *TyG* triglyceride-glucose index, *GTT* glucose tolerance test

Outcome ascertainment varied across studies. Self-reported, physician-diagnosed diabetes was the most common definition [19, 20, 22, 23, 26], whereas other studies used laboratory-based criteria, including fasting plasma glucose, glycated haemoglobin (HbA1c), and indices of insulin resistance such as HOMA-IR or the triglyceride–glucose (TyG) index [21, 24, 25, 27]. Most studies did not distinguish diabetes type; however, one cohort study specifically evaluated incident type 2 diabetes mellitus [28]. Most studies (8 of 10) defined mutually exclusive exposure categories, allowing separation of exclusive e-cigarette users from dual users (concurrent e-cigarette and combustible cigarette use). All studies relied on self-reported e-cigarette use; none employed biochemical verification of exposure status. Adjustment for potential confounders typically included age, sex, body mass index (BMI), race/ethnicity, income, education, physical activity, and alcohol consumption, although the specific covariates varied across studies.

Eight cross-sectional studies [19–24, 26, 27] reported adjusted odds ratios suitable for quantitative pooling. One cross-sectional study reported β coefficients from linear regression on log-transformed HOMA-IR and glucose tolerance test values [25], which were incompatible with odds ratios and therefore narratively synthesised. The sole prospective cohort study reported hazard ratios for incident type 2 diabetes [28] and was also narratively synthesised separately due to fundamental differences in study design and effect measure.

### Meta-analysis: prediabetes

**Fig. 2:**
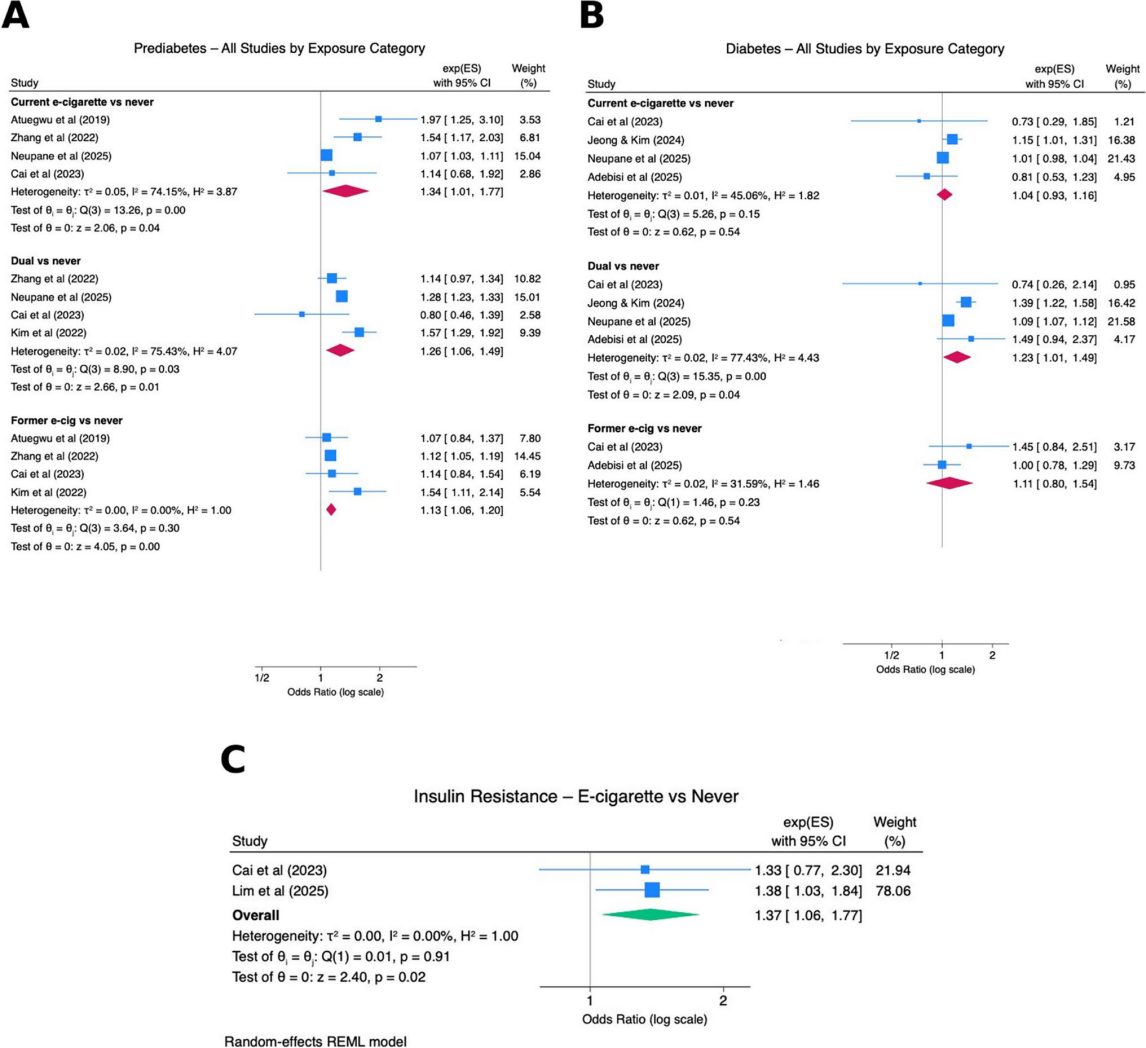
Forest plots of the association between e-cigarette use and glycaemic outcomes, stratified by exposure category (current exclusive e-cigarette users, dual users, and former e-cigarette users versus never users, where applicable). (**A**) Prediabetes. (**B**) Diabetes. (**C**) Insulin resistance (current exclusive e-cigarette users versus never users only). Effect estimates are expressed as odds ratios with 95% confidence intervals from random-effects (REML) meta-analysis

Twelve study-specific estimates from five studies contributed to the prediabetes meta-analysis (Fig. 2A). In subgroup analysis by exposure category, current exclusive e-cigarette users had a pooled OR of 1.34 (95% CI 1.01–1.77; *I*^2^ = 74.2%), dual users had a pooled OR of 1.26 (95% CI 1.06–1.49; *I*^2^ = 75.4%), and former e-cigarette users had a pooled OR of 1.13 (95% CI 1.06–1.20; *I*^2^ = 0%). All three subgroups demonstrated statistically significant associations.

### Meta-analysis: diabetes

Ten study-specific estimates from four studies contributed to the diabetes meta-analysis (Fig. 2B). Subgroup analysis revealed a heterogeneous pattern. Current exclusive e-cigarette users did not exhibit a statistically significant association with diabetes (OR 1.04; 95% CI 0.93–1.16; *I*^2^ = 45.1%). In contrast, dual users showed a significantly elevated pooled OR of 1.23 (95% CI 1.01–1.49; *I*^2^ = 77.4%). Former e-cigarette users showed a non-significant pooled OR of 1.11 (95% CI 0.80–1.54; *I*^2^ = 31.6%), though this subgroup was limited to two studies.

### Meta-analysis: insulin resistance

Two studies contributed to the insulin resistance meta-analysis (Fig. 2C). Both assessed current exclusive e-cigarette users versus never users. Cai et al. used HOMA-IR (upper tertile) in NHANES 2013–2018 (OR 1.33; 95% CI 0.77–2.30), while Lim et al. employed the TyG index in KNHANES 2019–2020 (OR 1.38; 95% CI 1.03–1.84). The pooled OR was 1.37 (95% CI 1.06–1.77), with no observed heterogeneity (*I*^2^ = 0%; *Q* = 0.01, *p* = 0.91). Given the small number of studies, this pooled estimate should be interpreted as exploratory.

A third study [25] also examined insulin resistance using HOMA-IR and glucose tolerance testing in NHANES 2013–2016 but reported β coefficients from linear regression on log-transformed outcomes, precluding inclusion in the pooled analysis. Sole e-cigarette users showed a non-significant positive coefficient for HOMA-IR (β 0.20; 95% CI −0.09 to 0.49) compared with non-users, while dual users showed no elevation (β − 0.13; 95% CI  − 0.43 to 0.16). These findings are not directly comparable due to different effect measures and outcome definitions.

### Prospective cohort evidence

The sole longitudinal study analysed 249,190 participants from the All of Us Research Program over a median follow-up of approximately 4 years [28]. Exclusive e-cigarette users did not demonstrate a significantly elevated risk of incident type 2 diabetes compared with never users (HR 0.88; 95% CI 0.66–1.16). By contrast, exclusive cigarette smokers had a significantly increased hazard (HR 1.18; 95% CI 1.11–1.26), and dual users showed a non-significant trend towards increased risk (HR 1.12; 95% CI 0.97–1.28).

### Risk of bias

The methodological quality of included studies was assessed using the Newcastle–Ottawa Scale (NOS), adapted for cross-sectional studies (maximum 10 stars) and in its standard form for the single cohort study (maximum 9 stars). All studies achieved NOS scores ≥ 7, indicating moderate to relatively high methodological quality within the constraints of observational designs.

Among the nine cross-sectional studies, scores ranged from 7 to 8 out of 10. Three studies [21, 25, 27] received the highest scores of 8, attributable to their use of laboratory-based outcome ascertainment (fasting plasma glucose, HbA1c, HOMA-IR, or TyG index), which warranted an additional star for objective outcome measurement. One study [24] also received a star for objective measurement (TyG index) but scored 7 overall owing to incomplete reporting of non-response characteristics. The remaining five cross-sectional studies [19, 20, 22, 23, 26] each scored 7, with points deducted for reliance on self-reported outcome ascertainment and self-reported exposure assessment.

The sole prospective cohort study [28] scored 7 out of 9. Strengths included its large and diverse sample drawn from the All of Us Research Program, appropriate exclusion of prevalent diabetes at baseline, adjustment for multiple confounders (receiving both comparability stars), outcome ascertainment via electronic health records, and adequate follow-up duration of approximately four years. Stars were not awarded for exposure ascertainment (self-reported, without biomarker verification) or adequacy of follow-up (attrition rate not fully reported).

The most pervasive methodological limitation across the entire evidence base was the universal reliance on self-reported e-cigarette use. No study employed biochemical verification of exposure status (e.g. urinary cotinine or exhaled carbon monoxide), introducing the possibility of exposure misclassification in all included analyses. Additionally, six of the nine cross-sectional studies ascertained diabetes or prediabetes status through self-reported physician diagnosis rather than standardised laboratory criteria, which may underestimate true prevalence and introduce differential misclassification if health-seeking behaviour varies by smoking or vaping status.

### Certainty of evidence (GRADE assessment)

The certainty of evidence was assessed using the Grading of Recommendations, Assessment, Development and Evaluations (GRADE) framework, applied to each outcome separately (Table 3). For observational studies, the starting certainty is low. For prediabetes, certainty was rated very low. The evidence was rated down for serious risk of bias (all cross-sectional designs with self-reported exposure and predominantly self-reported outcome ascertainment, precluding establishment of temporality and introducing susceptibility to reverse causation) and serious inconsistency (substantial heterogeneity within the current e-cigarette user [*I*^2^ = 74.2%] and dual user [*I*^2^ = 75.4%] subgroups, with point estimates ranging from 1.07 to 1.97 across individual studies). No concerns were identified for indirectness, or imprecision (the pooled confidence intervals excluded the null in all three subgroups).

**Table 3:** GRADE summary table

Outcome	No. of studies	Design	Risk of bias	Inconsistency	Indirectness	Imprecision	Publication bias	Overall certainty
Prediabetes	5 (cross-sectional)	Observational	Serious^a^	Serious^b^	Not serious	Not serious	Not assessable	⊕ ○○○ Very low
Diabetes	4 (cross-sectional) + 1 cohort^c^	Observational	Serious^a^	Serious^d^	Not serious	Serious^e^	Not assessable	⊕ ○○○ Very low
Insulin resistance	2 (cross-sectional)	Observational	Serious^a^	Not serious^f^	Serious^g^	Serious^h^	Not assessable	⊕ ○○○ Very low

^a^Cross-sectional, precluding temporal inference; all relied on self-reported e-cigarette use without biochemical verification; most ascertained outcomes via self-report

^b^I^2^ = 74.2% among current e-cigarette users and 75.4% among dual users; point estimates ranged from 1.07 to 1.97 across individual studies

^c^The prospective cohort study (Erhabor et al.) was narratively synthesised and reported no significant association for exclusive e-cigarette use (HR 0.88; 95% CI: 0.66–1.16)

^d^I^2^ = 77.4% among dual users; point estimates ranged from 0.73 to 1.49 across studies, with divergent directions of effect across subgroups

^e^Confidence intervals for current e-cigarette user (0.93–1.16) and former e-cigarette user (0.80–1.54) subgroups included the null

^f^I^2^ = 0%, but based on only two studies

^g^Studies used different insulin resistance indices (HOMA-IR and TyG index)

^h^Only two studies; insufficient to reliably assess consistency or publication bias

For diabetes, certainty was rated very low. The evidence was rated down for serious risk of bias (as above), serious inconsistency (*I*^2^ = 77.4% among dual users, with point estimates ranging from 0.73 to 1.49 across studies and divergent directions of effect across subgroups), and serious imprecision (confidence intervals for current and former e-cigarette user subgroups included the null). The sole prospective cohort study reported no significant association between exclusive e-cigarette use and incident type 2 diabetes. For insulin resistance, certainty was rated very low. The evidence was rated down for serious risk of bias (cross-sectional design and self-reported exposure), serious indirectness (the two pooled studies employed different indices—HOMA-IR and TyG index—which capture partially distinct aspects of insulin resistance), and serious imprecision (only two studies contributed, precluding reliable assessment of consistency and publication bias). Inconsistency was not downgraded (*I*^2^ = 0%), though this should be interpreted cautiously given the small number of studies.

Across all three outcomes, the universal reliance on self-reported e-cigarette use without biochemical verification and the predominance of cross-sectional designs were the most influential factors limiting certainty. The overall certainty for the association between e-cigarette use and glycaemic outcomes is very low.

## Discussion

This systematic review and meta-analysis brought together epidemiological evidence on the association between e-cigarette use and glycaemic outcomes across 10 observational studies involving more than 2.8 million participants. In cross-sectional analyses, e-cigarette users had higher pooled odds of prediabetes and insulin resistance than never users. For diabetes, however, the pattern varied according to exposure category. A statistically significant association was observed among dual users, whereas no significant association was found for current exclusive e-cigarette use. These findings should be interpreted with caution and do not support a straightforward causal reading. Within the cross-sectional evidence, current exclusive e-cigarette use was not significantly associated with diabetes, and heterogeneity in this subgroup was moderate and lower than that seen in other comparisons. Furthermore, the only prospective cohort study [28], which is more informative for temporal ordering and incident disease, also reported no association between exclusive e-cigarette use and incident type 2 diabetes. The convergence of these two null findings for the most clinically consequential outcome is central to interpreting this evidence base.

One explanation is that e-cigarette use may be associated with small early disturbances in glucose regulation that do not eventually translate into clinically diagnosed diabetes. It is also possible that some of the observed metabolic changes relate to weight gain after smoking cessation among people who move from combustible cigarettes to e-cigarettes. Smoking cessation is commonly followed by short-term increases in body weight, greater caloric intake, and compensatory behaviours such as snacking, all of which may temporarily worsen insulin resistance [11]. In this setting, any association between e-cigarette use and insulin resistance may partly reflect metabolic adjustment after smoking cessation rather than a direct effect of nicotine itself. Another explanation is methodological rather than biological. Prediabetes and insulin resistance often remain undetected, and many people with these conditions may not know that they have early metabolic impairment [29]. Consequently, they may be less likely to change their smoking or vaping behaviour. By contrast, a diagnosis of diabetes is a major clinical event and may prompt behavioural modification [30], including smoking cessation or a switch to e-cigarettes as a harm-reduction approach. This difference in health awareness could exaggerate cross-sectional associations for subclinical metabolic outcomes while leading to weaker or null associations for diagnosed diabetes, which is broadly in keeping with the pattern observed in this review.

The absence of an association between current exclusive e-cigarette use and diabetes is also supported by prospective evidence. Erhabor et al. [28], using data from nearly 250,000 participants followed for about 4 years, did not find an increased risk of incident type 2 diabetes among exclusive e-cigarette users. By comparison, exclusive cigarette smokers in the same study had a clearly higher risk. This contrast is informative because it shows that, within the same longitudinal setting, combustible cigarette smoking was associated with incident diabetes whereas exclusive e-cigarette use was not. The agreement between the cross-sectional null finding for current exclusive e-cigarette use and diabetes and the longitudinal null finding for incident type 2 diabetes strengthens the overall interpretation of the evidence. Taken together, the currently available observational literature does not indicate a clear independent association between exclusive e-cigarette use and clinical diabetes. Nevertheless, the prospective evidence has important limitations. It is derived from a single cohort study, exposure was self-reported, and the duration of follow-up was relatively short. A follow-up period of 4 years may not be long enough to capture progression from early metabolic disruption to diagnosed diabetes, particularly if any effect of e-cigarette use is small or accumulates over time. This point is relevant given that exclusive cigarette smokers in the same cohort showed a clearly elevated risk over that interval. In addition, the number of exclusive e-cigarette users was much smaller than the number of cigarette smokers, which may have reduced power to detect a modest association. For that reason, the null prospective finding is informative but should not be taken as conclusive evidence that no harm exists.

The positive cross-sectional associations observed for prediabetes and insulin resistance require cautious interpretation because the temporal sequence between exposure and outcome cannot be established [31]. Reverse causation therefore remains a credible explanation. People with diabetes, or those who perceive themselves to be at increased metabolic risk, may be more likely to stop smoking conventional cigarettes and switch to e-cigarettes as a harm-reduction option. In that case, e-cigarette use may reflect a response to existing metabolic concerns rather than a factor contributing to them, which could make cross-sectional estimates appear more suggestive of causality than is warranted.

The higher odds of prediabetes among former e-cigarette users are also compatible with this interpretation. Former users may include people who took up e-cigarettes during a period of health-related behavioural change and later stopped using them, while their underlying metabolic risk remained. Because prediabetes often goes undetected, these behavioural changes may take place without individuals knowing their metabolic status. Former e-cigarette use may therefore reflect reverse causation or clustering of pre-existing risk rather than a direct metabolic effect of past e-cigarette exposure.

Dual users were the only group with a statistically significant pooled association with diabetes, whereas no significant association was observed among current exclusive e-cigarette users. One possible explanation is that combined exposure to combustible cigarette smoke and e-cigarette aerosol may result in greater harm than exposure to e-cigarettes alone. However, residual confounding is also a strong possibility. Dual users frequently have higher nicotine dependence and a greater concentration of adverse health behaviours [32–34]. Compared with exclusive e-cigarette users or never users, they may be more likely to have lower socioeconomic status, poorer diet, lower levels of physical activity, and other clustered behavioural risks [34]. Although most of the included studies adjusted for important covariates, the adjustment strategies were not consistent across studies. Confounding by factors such as diet, genetic predisposition, psychosocial stress, sleep, and general health orientation cannot be ruled out. The lack of a significant association with diabetes among current exclusive e-cigarette users suggests that smoking-related exposure and correlated behavioural factors may explain a substantial part of the observed association among dual users.

Mechanistic evidence from preclinical studies suggests that smoking may disrupt pancreatic beta-cell function, stimulate sympathetic activity, and promote peripheral insulin resistance [35–37]. A biologically plausible pathway specific to e-cigarette exposure may involve nicotine itself, which has been shown experimentally to impair insulin signalling in skeletal muscle and other peripheral tissues, increase insulin resistance [45–47], and adversely affect pancreatic β-cell survival and mitochondrial function [48, 49]. These mechanisms may help explain why some cross-sectional studies observed associations with prediabetes or insulin resistance, even though the epidemiological evidence for overt diabetes remains weaker and currently non-causal. E-cigarette aerosol may also contain carbonyl compounds and reactive oxygen species with inflammatory and oxidative potential [38, 39]. In animal studies, chronic exposure has been linked to metabolic changes such as impaired glucose tolerance and hepatic steatosis [40].

The only included study that combined preclinical and epidemiological evidence reported that, in the animal component, exposure to e-cigarette aerosol was associated with impaired insulin-stimulated glucose uptake [25]. By contrast, the cross-sectional NHANES 2013–2016 analysis did not show a statistically significant association between exclusive e-cigarette use and HOMA-IR or glucose tolerance. Dual use was also not significantly associated with higher HOMA-IR. The contrast between these findings within the same study illustrates the difficulty of applying results from controlled animal exposure experiments to human populations and supports caution in drawing clinical conclusions from mechanistic data alone.

The extent to which these preclinical findings apply to human e-cigarette users is still unclear. Exposure conditions in animal studies may differ substantially from real-world patterns of use [41]. Differences between species in metabolic response are also relevant. In addition, concentrations of harmful constituents in e-cigarette aerosol are generally lower than those found in combustible cigarette smoke. This may partly explain why the epidemiological evidence, especially the prospective study and the diabetes findings among current exclusive users, does not clearly mirror the signals seen in preclinical work. Mechanistic plausibility is useful for generating hypotheses, but by itself it cannot demonstrate causation [42].

This review has several strengths. To our knowledge, it is the first meta-analysis to evaluate prediabetes, diabetes, and insulin resistance separately as related but distinct outcomes along the glycaemic continuum. Stratification by exposure category allowed a more informative interpretation than approaches that combine all e-cigarette users into a single group. This was particularly important in showing the null association between current exclusive e-cigarette use and diabetes, which may not have been apparent in unstratified analyses. Interpretation was also strengthened by the inclusion and close consideration of the only available prospective cohort study.

Several limitations should also be noted. Most notably, 9 of the 10 included studies were cross-sectional, limiting the ability to draw causal conclusions. Reverse causation and residual confounding therefore remain important concerns. E-cigarette exposure was based on self-report and was not biochemically verified, creating the possibility of misclassification. In addition, the reference groups were not consistent across studies: comparators included never users of e-cigarettes, sometimes restricted further to never smokers, as well as non-current users or broader categories such as “non-smoker/non-user,” depending on the dataset and analytic approach. This lack of uniformity may have reduced comparability across studies and may also have contributed to heterogeneity in the pooled estimates through differential misclassification. Outcome measurement also varied across studies, and several relied on self-reported physician diagnosis rather than standardised laboratory assessment, which may have introduced further misclassification and heterogeneity. Lastly, the meta-analysis of insulin resistance was based on only two studies, so that pooled estimate should be interpreted with particular caution. A further limitation is the heterogeneity of e-cigarette products. Included studies did not provide sufficiently detailed or standardised information on device type, power or temperature settings, nicotine formulation or concentration, e-liquid composition, or intensity of use. These factors may influence toxicant exposure and biological effects, but could not be evaluated in the present synthesis. Although the cumulative sample size was large, meta-analytic subgroup analyses by sex, age, body weight, or nicotine intake were not feasible because these data were not reported consistently across primary studies, individual participant data were unavailable, and no included study provided biomarker-based quantification of nicotine exposure.

These findings require careful interpretation. In cross-sectional analyses, current exclusive e-cigarette use and dual use were associated with higher odds of prevalent prediabetes and insulin resistance, suggesting a possible relationship with adverse glycaemic markers. The evidence for diabetes, however, is less convincing. Current exclusive e-cigarette use was not associated with prevalent diabetes in the cross-sectional analyses, and the only prospective cohort study found no association between exclusive e-cigarette use and incident type 2 diabetes. Considered together, this pattern does not justify strong causal inferences about diabetes risk based mainly on cross-sectional evidence. Clinicians may nevertheless consider monitoring glycaemic risk factors among e-cigarette users, especially dual users, while making clear that the findings differ according to both outcome definition and exposure category. Future research and surveillance should continue to distinguish exclusive e-cigarette use from dual use.

A key priority is the availability of additional large prospective cohort studies with longer follow-up. Such studies should incorporate objective exposure measures and standardised laboratory-based glycaemic outcomes. Mendelian randomisation approaches using variants related to nicotine metabolism may also help to address residual confounding. Future studies should also examine potential effect modification by device type, nicotine concentration, flavouring, intensity and duration of use, and baseline metabolic risk. Until stronger evidence becomes available, cross-sectional findings should be interpreted in light of their design limitations and considered alongside the currently limited prospective evidence.

## Conclusion

This systematic review and meta-analysis found differing patterns of association between e-cigarette use and glycaemic outcomes. Exclusive e-cigarette use was not associated with diabetes in either cross-sectional or prospective analyses, whereas dual use showed a statistically significant association that may be explained, at least in part, by concurrent combustible cigarette exposure and related behavioural risk factors. Cross-sectional analyses also indicated higher odds of prediabetes and insulin resistance among exclusive e-cigarette users, but these findings do not permit causal inference because of the potential for reverse causation and residual confounding. Additional large prospective studies using objective exposure biomarkers and standardised glycaemic outcomes are needed to clarify these relationships. Ongoing trials, including the DiaSmokeFree multicentre randomised controlled study [43], may help provide stronger evidence.

## Supplementary Information

Below is the link to the electronic supplementary material.

Supplementary file1 (PDF 177 KB)

## Data Availability

All data analysed during this study were extracted from previously published studies cited in the reference list. No new datasets were generated. Extracted data and analytic code are available from the corresponding author upon reasonable request.
