# Supplementary material for: Association between electronic cigarette use and prediabetes, diabetes, and insulin resistance: a systematic review and meta-analysis
Source: Intern Emerg Med. 2026 Jun 13;21(5):1879–93. doi: 10.1007/s11739-026-04430-x (PMC13452785; doi:10.1007/s11739-026-04430-x)
Supplement: Supplementary file 1 [file 11739_2026_4430_MOESM1_ESM.pdf]

PRISMA 2020 Checklist

Section and Topic	Item #	Checklist item	Location where item is reported
**TITLE**			
Title	1	Identify the report as a systematic review.	Title page
**ABSTRACT**			
Abstract	2	See the PRISMA 2020 for Abstracts checklist.	Abstract (page 2); Reporting follows the PRISMA 2020 for Abstracts checklist
**INTRODUCTION**			
Rationale	3	Describe the rationale for the review in the context of existing knowledge.	Section 1 (Introduction), paragraphs 1–3
Objectives	4	Provide an explicit statement of the objective(s) or question(s) the review addresses.	Section 1 (Introduction), final paragraph
**METHODS**			
Eligibility criteria	5	Specify the inclusion and exclusion criteria for the review and how studies were grouped for the syntheses.	Section 2.2 (Eligibility Criteria)
Information sources	6	Specify all databases, registers, websites, organisations, reference lists and other sources searched or consulted to identify studies. Specify the date when each source was last searched or consulted.	Section 2.1 (Search Strategy) — PubMed, Scopus, and Embase searched from database inception through December 2025
Search strategy	7	Present the full search strategies for all databases, registers and websites, including any filters and limits used.	Section 2.1 (Search Strategy); full strategies in Table 1
Selection process	8	Specify the methods used to decide whether a study met the inclusion criteria of the review, including how many reviewers screened each record and each report retrieved, whether they worked independently, and if applicable, details of automation tools used in the process.	Section 2.3 (Study Selection and Data Extraction) — two reviewers screened independently; discrepancies resolved by consensus with a third reviewer
Data collection process	9	Specify the methods used to collect data from reports, including how many reviewers collected data from each report, whether they worked independently, any processes for obtaining or confirming data from study investigators, and if applicable, details of automation tools used in the process.	Section 2.3 (Study Selection and Data Extraction) — standardised piloted data-extraction form

PRISMA 2020 Checklist

Section and Topic	Item #	Checklist item	Location where item is reported
			used by two reviewers
Data items	10a	List and define all outcomes for which data were sought. Specify whether all results that were compatible with each outcome domain in each study were sought (e.g. for all measures, time points, analyses), and if not, the methods used to decide which results to collect.	Section 2.2 (Eligibility Criteria) and Section 2.3 (Data Extraction) — outcomes: diabetes, prediabetes, insulin resistance
	10b	List and define all other variables for which data were sought (e.g. participant and intervention characteristics, funding sources). Describe any assumptions made about any missing or unclear information.	Section 2.3 (Study Selection and Data Extraction) — study characteristics, exposure categories, covariates, adjusted estimates
Study risk of bias assessment	11	Specify the methods used to assess risk of bias in the included studies, including details of the tool(s) used, how many reviewers assessed each study and whether they worked independently, and if applicable, details of automation tools used in the process.	Section 2.4 (Risk of Bias and Certainty of Evidence Assessment) — Newcastle–Ottawa Scale; two reviewers, independently
Effect measures	12	Specify for each outcome the effect measure(s) (e.g. risk ratio, mean difference) used in the synthesis or presentation of results.	Section 2.5 (Statistical Analysis) — adjusted odds ratios with 95% CIs; hazard ratio for the prospective cohort
Synthesis methods	13a	Describe the processes used to decide which studies were eligible for each synthesis (e.g. tabulating the study intervention characteristics and comparing against the planned groups for each synthesis (item #5)).	Section 2.5 (Statistical Analysis); Section 3.2 (Study Characteristics)
	13b	Describe any methods required to prepare the data for presentation or synthesis, such as handling of missing summary statistics, or data conversions.	Section 2.5 (Statistical Analysis) — effect estimates log-transformed for pooling and back-transformed for presentation
	13c	Describe any methods used to tabulate or visually display results of individual studies and syntheses.	Section 2.5 (Statistical Analysis);

Section and Topic	Item #	Checklist item	Location where item is reported
			Section 3 (Results); Figure 2 (forest plots); Tables 2–3
	13d	Describe any methods used to synthesize results and provide a rationale for the choice(s). If meta-analysis was performed, describe the model(s), method(s) to identify the presence and extent of statistical heterogeneity, and software package(s) used.	Section 2.5 (Statistical Analysis) — random-effects meta-analysis with REML estimation; I^2 and Cochran's Q for heterogeneity; Stata 18 (meta suite)
	13e	Describe any methods used to explore possible causes of heterogeneity among study results (e.g. subgroup analysis, meta-regression).	Section 2.5 (Statistical Analysis); Sections 3.3–3.5 — subgroup analyses by exposure category (current exclusive users, dual users, former users); Qb test for between-subgroup differences
	13f	Describe any sensitivity analyses conducted to assess robustness of the synthesized results.	Not applicable — sensitivity analyses were not feasible because each pooled comparison included fewer than 10 studies
Reporting bias assessment	14	Describe any methods used to assess risk of bias due to missing results in a synthesis (arising from reporting biases).	Section 2.5 (Statistical Analysis) — formal small-study/reporting bias assessment not performed (fewer than 10 studies per comparison)
Certainty assessment	15	Describe any methods used to assess certainty (or confidence) in the body of evidence for an outcome.	Section 2.4 (Risk of Bias and Certainty of Evidence Assessment) — GRADE framework applied across risk of bias, inconsistency,

Section and Topic	Item #	Checklist item	Location where item is reported
			indirectness, imprecision, and publication bias
**RESULTS**			
Study selection	16a	Describe the results of the search and selection process, from the number of records identified in the search to the number of studies included in the review, ideally using a flow diagram.	Section 3.1 (Study Selection); Figure 1 (PRISMA 2020 flow diagram)
	16b	Cite studies that might appear to meet the inclusion criteria, but which were excluded, and explain why they were excluded.	Section 3.1; full list of records assessed at full-text stage and reasons for exclusion available from the corresponding author on reasonable request
Study characteristics	17	Cite each included study and present its characteristics.	Section 3.2 (Study Characteristics); Table 2
Risk of bias in studies	18	Present assessments of risk of bias for each included study.	Section 3.7 (Risk of Bias)
Results of individual studies	19	For all outcomes, present, for each study: (a) summary statistics for each group (where appropriate) and (b) an effect estimate and its precision (e.g. confidence/credible interval), ideally using structured tables or plots.	Sections 3.3–3.6; Figure 2 (forest plots, panels A–C)
Results of syntheses	20a	For each synthesis, briefly summarise the characteristics and risk of bias among contributing studies.	Sections 3.3–3.6 (Meta-Analysis subsections); Section 3.7 (Risk of Bias)
	20b	Present results of all statistical syntheses conducted. If meta-analysis was done, present for each the summary estimate and its precision (e.g. confidence/credible interval) and measures of statistical heterogeneity. If comparing groups, describe the direction of the effect.	Sections 3.3–3.6; Figure 2 — pooled ORs with 95% CIs and I^2 values for each exposure subgroup and outcome
	20c	Present results of all investigations of possible causes of heterogeneity among study results.	Sections 3.3–3.5 — heterogeneity explored via subgroup analyses by exposure category

PRISMA 2020 Checklist

Section and Topic	Item #	Checklist item	Location where item is reported
	20d	Present results of all sensitivity analyses conducted to assess the robustness of the synthesized results.	Not applicable — sensitivity analyses not performed (see item 13f)
Reporting biases	21	Present assessments of risk of bias due to missing results (arising from reporting biases) for each synthesis assessed.	Not applicable — fewer than 10 studies per pooled comparison precluded formal assessment of small-study/reporting biases (see Section 2.5)
Certainty of evidence	22	Present assessments of certainty (or confidence) in the body of evidence for each outcome assessed.	Section 3.8 (Certainty of Evidence — GRADE Assessment); Table 3
**DISCUSSION**			
Discussion	23a	Provide a general interpretation of the results in the context of other evidence.	Section 4 (Discussion), paragraphs 1–6
	23b	Discuss any limitations of the evidence included in the review.	Section 4 (Discussion), limitations paragraph
	23c	Discuss any limitations of the review processes used.	Section 4 (Discussion), limitations paragraph
	23d	Discuss implications of the results for practice, policy, and future research.	Section 4 (Discussion), final paragraphs; Section 5 (Conclusion)
**OTHER INFORMATION**			
Registration and protocol	24a	Provide registration information for the review, including register name and registration number, or state that the review was not registered.	Section 2 (Methods) — this review was not prospectively registered in PROSPERO; acknowledged as a methodological limitation

PRISMA 2020 Checklist

Section and Topic	Item #	Checklist item	Location where item is reported
	24b	Indicate where the review protocol can be accessed, or state that a protocol was not prepared.	Not applicable — a formal protocol was not prepared
	24c	Describe and explain any amendments to information provided at registration or in the protocol.	Not applicable — no protocol or registration to amend
Support	25	Describe sources of financial or non-financial support for the review, and the role of the funders or sponsors in the review.	Declarations — Funding (Ministero dell'Università e della Ricerca, Bando PNRR 341/2022, Heal Italy CUP E63C22002080006). Funders had no role in the design, conduct, analysis, interpretation, or reporting of the review
Competing interests	26	Declare any competing interests of review authors.	Declarations — Competing interests
Availability of data, code and other materials	27	Report which of the following are publicly available and where they can be found: template data collection forms; data extracted from included studies; data used for all analyses; analytic code; any other materials used in the review.	Declarations — Availability of data and materials. Extracted data and analytic code available from the corresponding author on reasonable request; all primary studies are cited

From: Page MJ, McKenzie JE, Bossuyt PM, Boutron I, Hoffmann TC, Mulrow CD, et al. The PRISMA 2020 statement: an updated guideline for reporting systematic reviews. BMJ 2021;372:n71. doi: 10.1136/bmj.n71. This work is licensed under CC BY 4.0. To view a copy of this license, visit https://creativecommons.org/licenses/by/4.0/
